# Cord blood granulocyte levels are associated with severe bronchiolitis in the first year of life

**DOI:** 10.1002/cti2.70004

**Published:** 2024-09-25

**Authors:** Gabriela Martins Costa Gomes, Carla Rebeca Da Silva Sena, Vanessa E Murphy, Philip M Hansbro, Malcolm R Starkey, Peter G Gibson, Joerg Mattes, Adam M Collison

**Affiliations:** ^1^ Asthma and Breathing Program, Hunter Medical Research Institute The University of Newcastle Newcastle NSW Australia; ^2^ School of Medicine and Public Health The University of Newcastle Newcastle NSW Australia; ^3^ Centre for Inflammation Faculty of Science, School of Life Sciences, Centenary Institute and University of Technology Sydney Sydney NSW Australia; ^4^ Department of Immunology, School of Translational Medicine Monash University Melbourne VIC Australia; ^5^ Department of Respiratory and Sleep Medicine John Hunter Hospital Newcastle NSW Australia; ^6^ Paediatric Respiratory & Sleep Medicine Department John Hunter Children's Hospital Newcastle NSW Australia

**Keywords:** bronchiolitis, cord blood, eosinophil, neutrophil

## Abstract

**Objectives:**

Bronchiolitis is a leading cause of infant hospitalisation in the first year of life, and it preferentially affects infants born to mothers with asthma. Here, we evaluate cord blood granulocytes in infants born to mothers with asthma participating in the Breathing for Life Trial (BLT), to investigate early life determinants of bronchiolitis hospitalisation within the first year of life.

**Methods:**

Cord blood from 89 participants was collected into EDTA tubes and processed within 6 h of birth. Cells were stained in whole cord blood for eosinophils (CD45^+^, CD193^+^, CD16^−^), and neutrophils (CD45^+^, CD193^−^, CD16^+^). Medical records were reviewed for bronchiolitis hospitalisation in the first 12 months of life. Statistical analyses were conducted using Stata IC16.1.

**Results:**

Logistic regression adjusted for caesarean section, gestational age, maternal smoking during pregnancy, foetal heart deceleration during labour, and season of birth revealed an association between cord blood eosinophil levels and bronchiolitis hospitalisation in the first 12 months of life with an Area Under the Curve (AUC) of the Receiver Operating Characteristic (ROC) curve of 0.943 (aOR = 1.35, *P* = 0.011). Neutrophils were associated with the risk of bronchiolitis hospitalisation in a univariable logistic regression (OR = 0.93, *P* = 0.029); however, there was no statistical significance in the adjusted model.

**Conclusions:**

Higher eosinophil numbers in cord blood were associated with bronchiolitis hospitalisation in the first 12 months in a cohort of infants born to asthmatic mothers. This suggests that susceptibility to bronchiolitis in later life is influenced by the immune cell profile prior to viral infection.

## Graphical abstract

In this study, we found that higher eosinophil numbers in cord blood were associated with bronchiolitis hospitalisation in the first 12 months of life in a cohort of infants born to mothers with asthma. This suggests that susceptibility to bronchiolitis in later life may be influenced by the immune cell profile prior to viral infection.

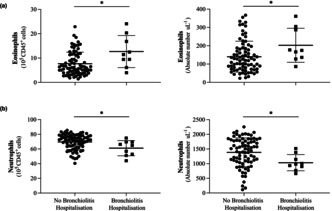

## Introduction

Bronchiolitis, a common respiratory condition in infants, remains one of the most common causes for infant hospitalisation across the first year of life. It is a leading cause of infant hospitalisation in high‐income countries.^1^, ^2^

A complex interaction between genes and environmental factors underpins the susceptibility to bronchiolitis in the first year of life, with a history of maternal asthma consistently identified as one of the strongest predictors.^3^, ^4^, ^5^ While improved asthma control in pregnancy has been shown to moderate this relationship,^6^ the underlying differences in the nascent immune system of infants who develop severe bronchiolitis compared to those who, when infected with the same respiratory virus, experience only mild symptoms, remains poorly understood. Identifying infants at high risk for hospitalisation based on clinical risk factors remains challenging.^7^ As a result, prophylaxis using existing single‐dose monoclonal antibodies is not deemed cost‐effective.^8^ While maternal vaccination for respiratory syncytial virus (RSV) may alleviate the disease burden in infants,^9^ it cannot address rhinovirus (RV) bronchiolitis, which is more strongly associated with preschool ventilation irregularities and an increased risk of subsequent asthma development^10^, ^11^ with children born to mothers with asthma during pregnancy at a particular risk.^12^

Granulocytes are essential effector cells involved in inflammation. Among them, neutrophils are the predominant immune cells in circulation and play a crucial role in the initial stages of the innate immune response against respiratory viral infections.^13^ Eosinophils are found in the airways and play a role in maintaining lung immune homeostasis under normal conditions. In the context of viral infections, eosinophils contribute to antiviral immune responses through the production of various soluble mediators.^14^ Their granules contain molecules with potential antiviral activity, and they can exert beneficial effects against respiratory viruses.^15^ Recently, the role of type 2 innate immune cells such as innate lymphoid cells type 2 (ILC2s) and eosinophils have also been shown to play key roles in early lung development which may precede susceptibility to severe bronchiolitis.^3^, ^16^, ^17^

Considering the direct impact of the maternal immune response at the foetal–maternal interface, it is important to note that neutrophils are among the innate immune cells present at this interface.^18^ Additionally, the release of type 2 cytokines by the mother during pregnancy may promote foetal eosinophilia, which can enhance airway sensory innervation.^3^ Here, we studied the aberrant innate immune response underpinning bronchiolitis by investigating whether differences in cord blood granulocyte populations precede bronchiolitis in the first year of life in infants born to mothers with asthma.

## Results

### Study population

To explore the associations between granulocyte profiles in cord blood and bronchiolitis occurrence in the initial year of life, infants born to asthmatic mothers participating in the Breathing for Life Trial (BLT)^19^ were included. Characteristics of the enrolled subjects are provided in Table 1. All participants provided written informed consent for this study that was approved by the Hunter New England Human Research Ethics Committee (2019/ETH03856). Between March 2017 and November 2019, 280 eligible infants were born to mothers participating in BLT at John Hunter Hospital, Newcastle site. From those infants, 89 (31.8%) had cord blood collected immediately after birth, and their cells were stained in whole cord blood for neutrophils and eosinophils based on specific surface markers (Supplementary table 1) within 6 h. Trained staff extracted information from medical records, revealing that 10.1% of these recruited babies were hospitalised for bronchiolitis. The distribution of the population was maintained as shown in Table 1.

**Table 1:** Characteristics of subjects with collected cord blood, along with those who had cord blood collected and experienced hospitalisation because of bronchiolitis

	All participants (*n* = 89)	No bronchiolitis hospitalisation (*n* = 80)	Bronchiolitis hospitalisation (*n* = 9)	*P*‐value
Maternal age at delivery, years *mean* (SD)	30.2 (5.4)	30.3 (5.4)	29.8 (5.2)	0.789
Gestational age, weeks *mean* (SD)	39.2 (1.3)	39.3 (1.3)	38.4 (1.2)	**0.050**
Birth weight, g *mean* (SD)	3507 (531.2)	3531.4 (501.5)	3315 (732.5)	0.104
Birth length, cm *mean* (SD)	51.4 (3.6)	51.6 (3.4)	49.5 (4.6)	0.087
Maternal smoking during pregnancy *n* (%)	11 (12.4)	5 (6.3)	6 (66.7)	**0.000**
Prematurity *n* (%)	1 (1.1)	1 (1.3)	0 (0.0)	0.736
Male *n* (%)	46 (51.7)	41 (51.3)	5 (55.6)	0.806
Foetal heart rate deceleration during labour *n* (%)	29 (32.6)	26 (32.5)	3 (33.3)	0.960
Delivery type				
Spontaneous *n (%)*	58 (65.2)	53 (66.3)	5 (55.6)	0.523
C‐section *n (%)*	31 (34.8)	27 (33.7)	4 (44.4)	
Season of birth				
Winter *n* (%)	25 (28.1)	23 (28.8)	2 (22.2)	0.841
Spring *n* (%)	26 (29.2)	24 (30.0)	2 (22.2)	
Summer *n* (%)	18 (20.2)	16 (20.0)	2 (22.2)	
Autumn *n* (%)	20 (22.5)	17 (21.3)	3 (33.3)	
Age at bronchiolitis hospitalisation, months *mean* (SD)	—	—	6.4 (2.6)	—
Length of hospital stay, days *mean* (SD)	—	—	2.6 (1.9)	—
High‐flow oxygen therapy *n* (%)	—	—	3 (33.3)	—
Salbutamol treatment *n* (%)	—	—	1 (11.1)	—

Groups were compared using either a *t*‐test or a Chi‐squared test as appropriate. *P*‐values < 0.05 in bold.

### Cord blood eosinophils and neutrophils are associated with hospitalisation because of bronchiolitis

The Mann–Whitney *U*‐test was used to investigate differences in mean cord blood eosinophil and neutrophil counts between infants hospitalised for bronchiolitis in their first year of life and those who were not.

**Figure 1:**
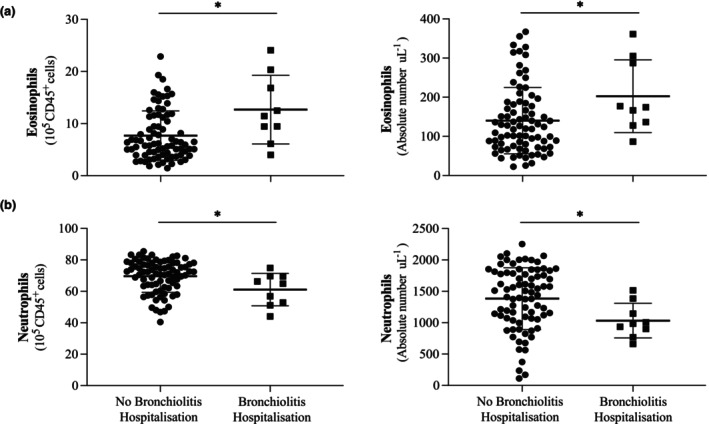
**(a)** Cord blood eosinophils normalised by CD45^+^ cells as well as in absolute numbers are elevated in infants experiencing hospitalisation because of bronchiolitis. **(b)** Cord blood neutrophils normalised by CD45^+^ cells as well as in absolute numbers are reduced in infants hospitalised because of bronchiolitis. Each data point represents a single participant, and bars show the median and standard deviation. Statistical significance was assessed using the Mann–Whitney *U*‐test. *P ≤ 0.05.

Results showed that infants hospitalised for bronchiolitis in their first year had elevated cord blood eosinophil counts, both normalised by CD45^+^ cells (*P* = 0.015) and in absolute numbers (*P* = 0.018, Figure 1a). Conversely, cord blood neutrophil counts were reduced in these infants, both normalised by CD45^+^ cells (*P* = 0.042) and in absolute numbers (*P* = 0.015, Figure 1b).

### Cord blood eosinophils and neutrophils are associated with subsequent hospital admission for bronchiolitis

Logistic regression analyses were performed to investigate the association between eosinophil and neutrophil counts in cord blood and subsequent hospitalisation because of bronchiolitis in the first year of life.

In a univariable logistic regression analysis, eosinophils (odds ratio (OR) = 1.17, 95% confidence interval (CI) 1.03 to 1.33, *P* = 0.011) and neutrophils (OR = 0.93, 95% CI 0.87 to 0.99, *P* = 0.029) were associated with the risk of hospitalisation when normalised by CD45^+^ cells. In absolute numbers, eosinophils had an OR = 1.01, 95% CI 1.00 to 1.01, *P* = 0.051, and neutrophils an OR = 1.00, 95% CI 0.99 to 1.00, *P* = 0.048 (Table 2).

**Table 2:** Univariable logistic regression and multivariable logistic regression to assess the risk for hospitalisation because of bronchiolitis in BLT study infants

	Univariable analysis (*n* = 89 total, *n* = 9 hospitalisations)		Multivariable analysis^a^ (*n* = 89 total, *n* = 9 hospitalisations)		
	OR (95% CI)	*P*‐value	aOR (95% CI)	*P*‐value	AUC ROC
**Eosinophils** ^**b**^					
Per CD45^+^	1.17 (1.03 to 1.33)	**0.011**	1.35 (1.07 to 1.70)	**0.011**	0.943
Per μL	1.01 (1.00–1.01)	0.051	1.02 (1.00–1.03)	**0.024**	0.935
**Neutrophils** ^**b**^					
Per CD45^+^	0.93 (0.87 to 0.99)	**0.029**	0.92 (0.83 to 1.01)	0.077	0.924
Per μL	1.00 (0.99 to 1.00)	**0.048**	1.00 (0.99 to 1.00)	0.099	0.918

aOR, adjusted odds ratio; AUC, area under the curve; BLT, breathing for life trial; CI, confidence interval; ROC, receiver operating characteristic.

*P*‐values < 0.05 in bold.

^a^ Multivariable analysis adjusted for caesarean section, gestational age, maternal smoking during pregnancy, foetal heart deceleration during labour, season of birth.

^b^ Results are expressed in 10^5^ of CD45^+^ cells.

In a multivariable logistic analysis, cord blood eosinophils were associated with bronchiolitis hospitalisation both normalised by CD45^+^ cells (aOR = 1.35, 95% CI 1.07 to 1.70, *P* = 0.011) and in absolute numbers (aOR = 1.02, 95% CI 1.00 to 1.03, *P* = 0.024), with an Area Under the Curve (AUC) of the Receiver Operating Characteristic (ROC) curve of 0.943 and 0.935 (respectively, Table 2, Supplementary figure 2a) with an optimal cut point sensitivity of 66.7% and 96.3% specificity. Maternal smoking during pregnancy was also positively associated with bronchiolitis risk (normalised by CD45^+^ cells: aOR = 99.14, 95% CI 6.37 to 1543.7, *P* = 0.001; absolute numbers: aOR = 72.09, 95% CI 6.02 to 863.81, *P* = 0.001).

There was no statistical significance of cord blood neutrophils in the adjusted model in infants with risk of hospitalisation because of bronchiolitis both normalised by CD45^+^ cells (aOR = 0.92, 95% CI 0.83 to 1.01, *P* = 0.077) and in absolute numbers (aOR = 1.00, 95%CI 0.99 to 1.00, *P* = 0.099), with an AUCROC of 0.924 and 0.918, respectively (Table 2, Supplementary figure 2b) and optimal cut point 66.7% sensitivity, 96.3% specificity. As expected, maternal smoking during pregnancy was again positively associated with bronchiolitis risk (normalised by CD45^+^ cells: aOR = 29.78, 95% CI 4.35 to 203.71, *P* = 0.001; absolute numbers: aOR = 32.96, 95% CI 4.76 to 228.2, *P* = 0.000).

### Cord blood eosinophils and neutrophils predict the number of hospitalisation days because of bronchiolitis

Linear regression analysis was conducted to explore the relationship between eosinophils or neutrophils in cord blood and the number of days hospitalised with bronchiolitis. Multivariable linear regression adjusted for the same confounders as previously; cord blood eosinophils were significantly associated with the days hospitalised with bronchiolitis (normalised by CD45^+^ cells: Coefficient 0.07, 95% CI 0.03 to 0.10, *P* = 0.001; absolute numbers: Coefficient 0.003, 95% CI 0.001 to 0.006, *P* = 0.005); and neutrophils were significantly associated with the days hospitalised with bronchiolitis when normalised by CD45^+^ cells (Coefficient − 0.03, 95% CI –0.05 to −0.01, *P* = 0.003) but not in absolute numbers (Coefficient − 0.0004, 95% CI –0.0009 to −0.00001, *P* = 0.056) (Table 3).

**Table 3:** Linear regression analysis to assess the association between granulocytes and days hospitalised with bronchiolitis in BLT study infants

	Univariable analysis (*n* = 89 total, *n* = 9 hospitalisations)		Multivariable analysis^a^ (*n* = 89 total, *n* = 9 hospitalisations)	
	Coefficient (95% CI)	*P*‐value	Coefficient (95% CI)	*P*‐value
**Eosinophils** ^b^				
Per CD45^+^	0.06 (0.02 to 0.10)	**0.001**	0.07 (0.03 to 0.10)	**0.001**
Per μL	0.003 (0.001 to 0.005)	**0.009**	0.003 (0.001 to 0.006)	**0.005**
**Neutrophils** ^b^				
Per CD45^+^	−0.03 (−0.05 to −0.01)	**0.002**	−0.03 (−0.05 to −0.01)	**0.003**
Per μL	−0.0004 (−0.0009 to –0.00003)	**0.036**	−0.0004 (−0.0009 to 0.00001)	0.056

BLT, breathing for life trial; CI, confidence interval.

*P*‐values < 0.05 in bold.

^a^ Multivariable analysis adjusted for caesarean section, gestational age, maternal smoking during pregnancy, foetal heart deceleration during labour, season of birth.

^b^ Results are expressed in 10^5^ of CD45^+^ cells.

## Discussion

While infection with RSV or RV is ubiquitous by 2 years of age, only a minority of infants experience bronchiolitis. Here, for the first time, we have explored the relationship between the granulocytes in cord blood, and the subsequent risk of bronchiolitis across the first 12 months. As bronchiolitis typically presents in otherwise well infants this poses logistical challenges for the prospective collection of data, particularly with the labour‐intensive nature of fluorescence‐activated cell sorting (FACS) immunophenotyping of cord blood. Our analysis of 89 infants in a prospective study design identified strong associations between eosinophil levels and bronchiolitis hospitalisations in the first year of life (Table 2).

Eosinophils, recognised as canonical type‐2 (T2) immune cells, are not commonly thought to play a principal role in the innate antiviral response. However, our findings reveal elevated eosinophil levels in the cord blood of infants at a heightened risk of bronchiolitis hospitalisation. During pregnancy, the maternal immune system is skewed towards T2 predominant responses to promote immune tolerance in the developing fetus. Interleukin (IL)‐33, known for promoting a T2 immune response, acts directly on eosinophils and regulates their biology.^20^ Studies show that IL‐33 regulates eosinophil survival^21^ and mediates the activation of ILC2s in the lungs,^22^ which has been shown to play key roles in early lung development^16^ and may precede susceptibility to severe bronchiolitis. Recent studies have also demonstrated that the canonical T2 cytokine, IL‐5, crosses the placental barrier and influences lung development. In mouse models, mothers with elevated IL‐5 mediate a foetal eosinophilia, resulting in pups with increased airway hyperreactivity and persistent airway epithelial nerve density into adulthood.^17^ This description of the developmental origins of airway disease aligns closely with the association in our cohort, where foetal cord blood eosinophils are associated with an elevated likelihood of severe bronchiolitis in the first year. This concept is further supported by previous human studies, which identified that infants with low circulating eosinophil levels during hospital admission for bronchiolitis had a significantly reduced risk of having an asthma diagnosis up to 31 years of age compared to infants with high eosinophil levels.^23^ Elevated eosinophil levels in the blood of infants admitted with bronchiolitis have also been demonstrated to be negatively associated with adult lung function at 17–20 years, assessed by forced vital capacity (FVC) and forced expiratory volume (FEV_1_) irrespective of the underlying pathogen, although most cases in this study were non‐RSV bronchiolitis. Interestingly, the levels of blood eosinophils during bronchiolitis were also negatively associated with non‐atopic asthma in early adulthood.^24^ In murine models of rhinoviral infections, eosinophils have been observed to downregulate the antiviral action of plasmacytoid dendritic cells, which was reversed with anti‐IL‐5 therapy.^25^, ^26^ Together, there is emerging evidence that eosinophils play an important immunoregulatory role in respiratory viral infections in general and severe bronchiolitis in particular.

The recruitment of neutrophils to the lung is well described in all viral causes of bronchiolitis, however, whether neutrophilic inflammation is of net benefit to the host remains uncertain.^13^ RSV‐induced bronchiolitis is the leading cause of hospital admission and death in infants.^27^ Previous studies have implicated neutrophils in epithelial damage associated with RSV infections,^28^ and mouse RSV models showed that depleting neutrophils had no impact on viral replication or disease severity.^29^ Decreased neutrophils in the cord blood were associated with the number of days hospitalised with bronchiolitis (Table 3), although they were not significantly associated with bronchiolitis hospitalisation once adjusted for confounders (Table 2). This may suggest that neutrophils play a beneficial role in viral clearance that mitigate the length of infection. In comparison, the immune predisposition marked by elevated eosinophils in the cord blood conveys an additional risk of infection itself, which also associates with increased days in hospital (Table 3). Further mechanistic studies are necessary to confirm this hypothesis.

A common limitation in studies using cord blood is the restricted sample size. However, the advantages of accessing a suitable quantity of blood so early in life have allowed studies with relatively small sample sizes to make significant contributions to our understanding of early life immune and respiratory development. Another limitation is that our population in the BLT study included only infants born to asthmatic mothers, so these results might differ in a non‐asthmatic population. However, it is worth noting that the majority of bronchiolitis hospitalisations occur in infants with no known risk factors.^30^ Our findings suggest that cord blood eosinophil levels demonstrate high sensitivity in identifying infants at risk of hospitalisation because of bronchiolitis and show specificity in correctly identifying those not at risk, with a relatively low rate of false positives. This indicates the potential utility of cord blood eosinophil measurements as predictive markers for bronchiolitis hospitalisation while both elevated eosinophils and decreased neutrophils predict more severe bronchiolitis requiring more hospitalisation days. The AUC is encouraging, however, given the limited number of cases, caution should be exercised in interpreting the cut points for sensitivity and specificity with these results only showing potential for a predictive measure for bronchiolitis. Further validation in larger cohorts, including infants born to mothers with no asthma, is essential to establish the reliability of these initial findings. If confirmed, this predictive tool could facilitate targeted prophylactic antibody treatments for high‐risk infants, addressing the current cost‐effectiveness challenges in broader population implementation.^7^ These data suggest the aberrant innate immune response to respiratory viral infection, which underlies bronchiolitis, may originate during *in utero* development, preceding exposure to the early life environment, which may open further opportunities for effective bronchiolitis prevention strategies through pregnancy.

## Methods

### Study design and participants

Pregnant asthmatic women, 18 years or older, with asthma diagnosed by a physician, and symptoms of asthma or use of asthma therapy (β2‐agonist, ICS) in the past 12 months, and who were 12–23 weeks' gestation (supported by ultra‐ sound or clinical obstetric assessment), were enrolled in the Breathing for Life Trial (BLT).^19^ The BLT is a multicentre (Brisbane (QLD), Canberra (ACT), Newcastle (NSW) and Sydney (NSW)) randomised controlled trial of asthma with prospective infant follow‐up. Drug or alcohol dependence, chronic oral corticosteroid use, chronic lung disease other than asthma, concomitant chronic illness were considered exclusion criteria. Mothers eligible for participation in the infant follow‐up within the BLT cohort at Newcastle, who provided consent, underwent cord blood collection after their baby's birth (*n* = 89). Additionally, medical records of infants who were hospitalised within the initial 12 months of life were reviewed. Bronchiolitis cases in this study were classified based on the final diagnosis provided by the clinical staff on the date of hospitalisation. Exclusion criteria for infants included the inability to collect the required amount of cord blood at birth and a lack of available clinical data on bronchiolitis status.

### Ethics statement

This research was approved by the Hunter New England Human Research Ethics Committee of the Hunter New England Local Health District (2019/ETH03856) and all women provided written informed consent before participation.

### Cord blood collection

Cord blood samples from BLT participants were collected at John Hunter Hospital (New South Wales, Australia) immediately after birth by needle puncture of the umbilical vein after the umbilical cord was detached from the infant. All samples were transferred into EDTA tubes to be stained by a trained staff within 6 h.

### Flow cytometry

Cord blood cells were stained in whole blood (300 μL total blood per sample) within 6 h and subsets were predefined based on specific surface markers for Eosinophils (CD45^+^, CD193^+^, CD16^−^), and neutrophils (CD45^+^, CD193^−^, CD16^+^) (Supplementary table 1, Supplementary figure 1). After 30 min of incubation, red blood cells were lysed using BD FACS™ Lysing Solution (BD Biosciences, San Jose, CA, USA) and washed. Samples were stored at 4°C and acquired within 48 h on LSRFortessa X‐20 flow cytometer (BD Biosciences, San Diego, CA, USA). The maximum number of events from each sample were acquired and recorded for each subject. Analyses of cell types were conducted with FlowJo software (v 10.5 – Flow Jo LLC, Ashland, OR, USA) for all cell populations. Results are shown as the total positive cells as a proportion of 10^5^ CD45^+^ cells, and absolute numbers calculated based on the total cells acquired and the percentage of positive cells.

### Statistical analysis

The Mann–Whitney *U*‐test was performed using GraphPad Prism software version 10.1.1 (GraphPad Software LLC, San Diego, CA, USA). Regression analyses were performed using Stata IC 16.1 (Stata Corporation, College Station, TX, USA). The influence of confounders was assessed by performing uni‐ and multivariable regression analysis. In multivariable analyses performed to identify variables associated with bronchiolitis hospitalisation, the following known confounders were included: (1) caesarean section, (2) gestational age, (3) maternal smoking during pregnancy, (4) season of birth and (5) foetal heart deceleration during labour (representing stress during delivery which tends to increase cord blood cell numbers).^31^ For all analyses, statistical significance was considered when *P* < 0.05.

## Author contributions

**Gabriela Martins Costa Gomes:** Data curation; formal analysis; investigation; methodology; visualization; writing – original draft; writing – review and editing. **Carla Rebeca Da Silva Sena:** Data curation; investigation; writing – review and editing. **Vanessa E Murphy:** Data curation; funding acquisition; investigation; project administration; writing – review and editing. **Philip M Hansbro:** Methodology; visualization; writing – review and editing. **Malcolm R Starkey:** Methodology; visualization; writing – review and editing. **Peter G Gibson:** Conceptualization; data curation; funding acquisition; investigation; project administration; writing – review and editing. **Joerg Mattes:** Conceptualization; data curation; funding acquisition; investigation; project administration; writing – review and editing. **Adam M Collison:** Conceptualization; data curation; formal analysis; funding acquisition; investigation; methodology; project administration; writing – original draft; writing – review and editing.

## Conflict of interest

The authors declare no conflict of interest.

## Supporting information


Supporting Information

## Data Availability

The data that support the findings of this study are available from the corresponding author upon reasonable request.
