# Supplementary material for: Cord blood granulocyte levels are associated with severe bronchiolitis in the first year of life
Source: Clin Transl Immunology. 2024 Sep 25;13(9):e70004. doi: 10.1002/cti2.70004 (PMC11424167; doi:10.1002/cti2.70004)

Title: Cord blood granulocyte levels are associated with severe bronchiolitis in the first year of life

Gabriela Martins Costa Gomes[1,2], Carla Rebeca Da Silva Sena[1,2], Vanessa E Murphy[1,2], Philip M Hansbro[3], Malcolm R Starkey[4], Peter G Gibson[1,5], Joerg Mattes[1,2,6], Adam M Collison[1,2#]

Supporting information

Supplementary table 1. Antibodies used for flow cytometry analysis.

CD-Antigen	Other Names	Isotope	Clone	Company	Brief Description
CD16	FcγRIIIA	BV421	3G8	BD bioscience	Low affinity Fcγ receptor, mediates phagocytosis and ADCC, degranulation
CD45	LCA	APC	HI30	BD bioscience	Activation, signalling
CD193	CCR3	PE	5E8	BD bioscience	Leukocytes chemotaxis, HIV-1 coreceptor

Supplementary figure 1. Flow cytometry gating strategy. Cord blood samples stained and acquired with a LSRFortessa X-20 flow cytometer and analysed using FlowJo software. Subsets were predefined based on specific surface markers for eosinophils ($CD45^{+}$, $CD193^{+}$, $CD16^{-}$) and neutrophils ($CD45^{+}$, $CD193^{-}$, $CD16^{+}$).

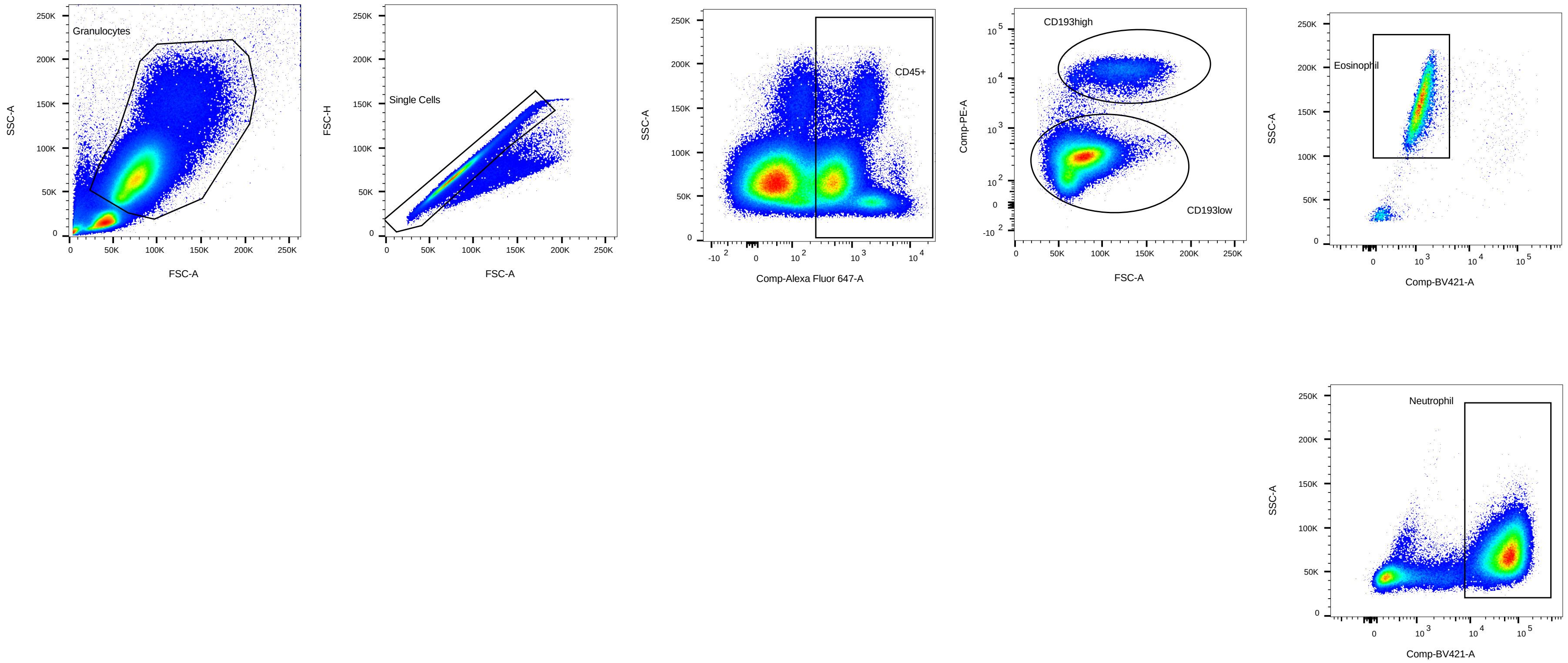

Supplementary figure 2. Receiver operating characteristic curve (ROC) for predicting hospitalisation due to bronchiolitis in BLT study infants within the first 12 months of life. **(a)** Using cord blood eosinophils, the Area Under the Curve (AUC) of the ROC for hospitalisations due to bronchiolitis is 0.943 when normalised by $CD45^+$ cells and 0.935 when considering absolute numbers. **(b)** For cord blood neutrophils, the AUC ROC for hospitalisations due to bronchiolitis is 0.924 when normalised by $CD45^+$ cells and 0.918 when considering absolute numbers.

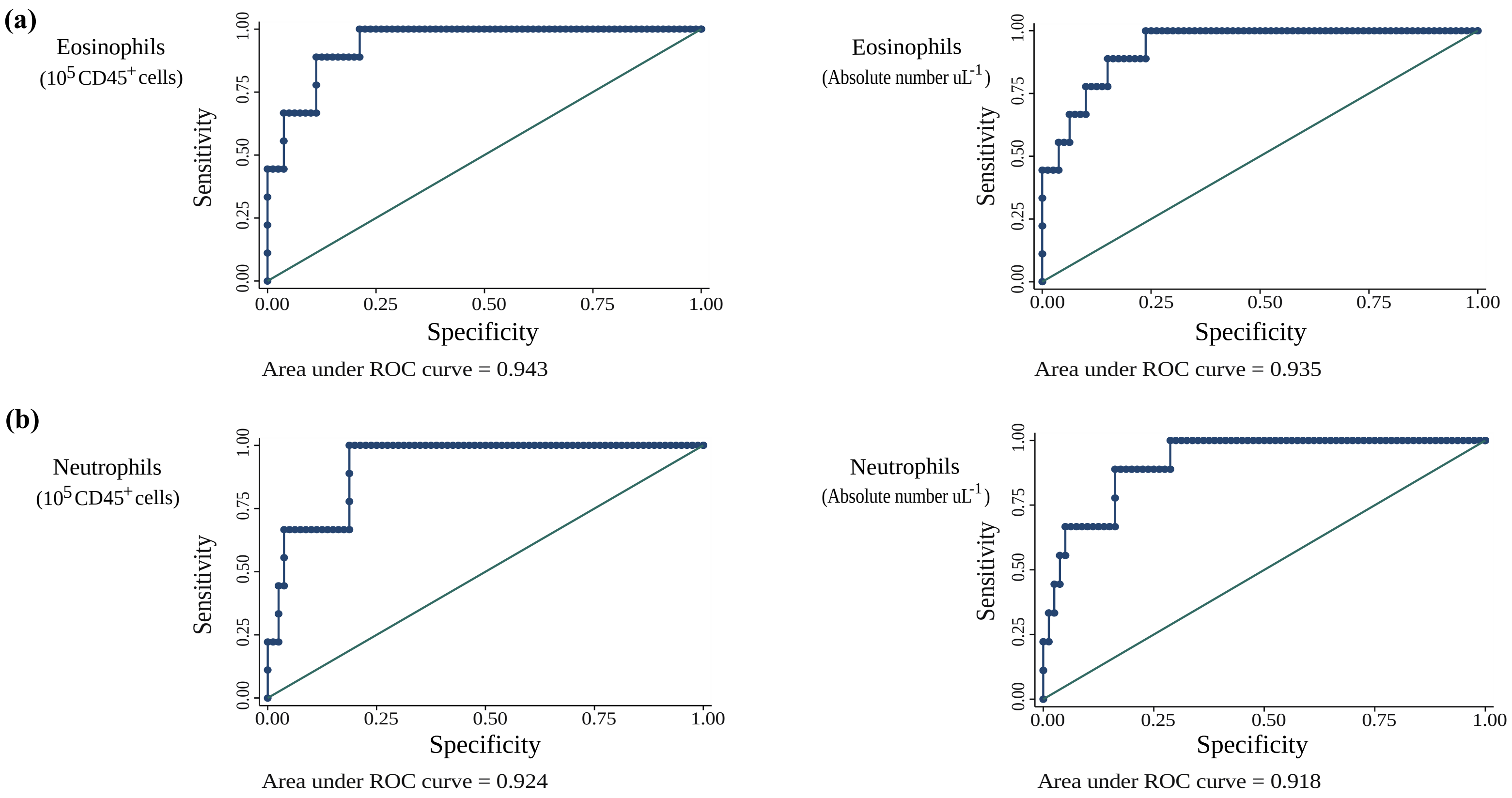

Supplement: Supplementary file 1 — Supporting Information [file CTI2-13-e70004-s001.pdf]
